# Ultrasonography in Acute Kidney Injury

**DOI:** 10.24908/pocus.v7iKidney.14989

**Published:** 2022-02-01

**Authors:** Andrew A Moses, Hilda E Fernandez

**Affiliations:** 1 Division of Nephrology, Department of Medicine, Vagelos College of Physicians and Surgeons, Columbia University New York, NY

**Keywords:** Ultrasound, POCUS, Acute kidney injury, Point-of-care ultrasound, nephrology

## Introduction

Advances in the use of ultrasonography can enhance our ability to better characterize acute kidney injury (AKI). AKI is a clinical syndrome characterized by a rapid decrease in kidney excretory function with the accumulation of products of nitrogen metabolism and other clinically unmeasured waste products, and may proceed to include clinical manifestations including decreased urine output, development of metabolic acidosis, and electrolyte abnormalities 1. The Kidney Disease Improving Global Outcomes (KDIGO) criteria defines AKI (Table 1). Staging severity of AKI guides the physician in respect to medical management and prognosis. The overall incidence of AKI is around 20% of patients hospitalized worldwide, and around 50% in intensive care unit (ICU) patients 2, 3. AKI has been found to have increasing morbidity and mortality, no matter the cause of admission, as well as an in-hospital mortality of close to 50% 4. In a large study of 8 ICUs over 8 years, Kellum et al. found that AKI was associated with increasing mortality rate with worsening AKI stage. A decrease in urine output alone, without an increase in serum creatinine, was associated with decreased 1-year survival 5. Recurrent AKI has also been associated with increased mortality, further demonstrating the importance of detecting, monitoring, and diagnosing AKI 6. 

**Table 1:** Stages of AKI as defined by KDIGO. (Adapted from 1.)

**Stage of AKI**	**Serum Creatinine**	**Urine output**
1	1.5-1.9 times baseline OR ≥0.3 mg/dL	<0.5mL/kg/h for 6-12 hours
2	2.0-2.9 times baseline	<0.5mL/kg/h for ≥12 hours
3	3.0 times baseline OR Increase in serum creatinine to ≥4.0 mg/dL OR initiation of kidney replacement therapy OR in patients < 18 years, decrease in GFR to <35 mL/min per 1.73 m^2^	<0.3 mL/kg/h for ≥24 hours OR Anuria for ≥12 hours

An approach to AKI differentiates between pre-renal, intrinsic, and post-renal/obstructive AKI. Pre-renal AKI results from decreased perfusion of the kidney parenchyma. Intrinsic AKI is an insult that progresses to damage of the kidney tubules and/or direct damage to the nephron itself. Post-renal AKI develops from an obstruction of urine downstream of the kidney collecting system, such as in nephrolithiasis, a malignancy compressing a ureter, or in prostatic hypertrophy. Pre-renal and post-renal injuries are typically reversible, but prolonged injury may develop into intrinsic AKI. The rapid diagnosis of pre- and post-renal obstruction can lead to reversal. Ultrasonography through high quality imaging is therefore critical for clinical care 7. Kidney ultrasonography is usually performed by trained ultrasound technicians with a radiologist interpretation. Point of care ultrasound, when wielded by a clinician, can aid in rapid evaluation of AKI. In this review we will examine the evolving role of kidney ultrasound in AKI management.

## Kidney Imaging

Kidney length norms were established by Emamian et al. who studied 665 healthy volunteers of various ages to establish median kidney lengths of 11.2 cm on the left and 10.9 on the right side 8. Kidney length correlates linearly with patient height, and so should be taken into account when determining expected kidney length 8. When performing kidney ultrasonography, taking the time to obtain multiple kidney images can assist in obtaining accurate measurements. In addition, be aware that congenital abnormalities of the kidney and urinary tract (CAKUT) have a prevalence of three and six per 1,000 live births 9, 10. Solitary kidney/unilateral kidney agenesis occurs in 1 in 1000 live births, more frequently in women and more often on the left 11. These patients have an increased risk of CKD progression 12. Duplicated collecting systems can also be observed in the absence of history of urinary tract infections and should be differentiated from hydronephrosis. Bladder imaging is important when performing kidney ultrasonography to identify the site of outflow obstruction.

**Figure 1:**
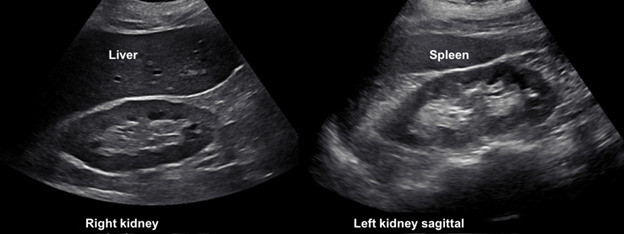
Normal kidney on ultrasound. Sagittal view of normal left and right kidney. Note the isoechoic nature of the cortex with the liver and spleen with hyperechoic sinus fat in the center. The hypoechoic medullary pyramids surround the sinus fat. Image from Koratala, A. POCUS Gallery. Renal Fellow Network. Accessed November 27, 2020. https://www.renalfellow.org/pocus-gallery/ Used with Permission

**Figure 2:**
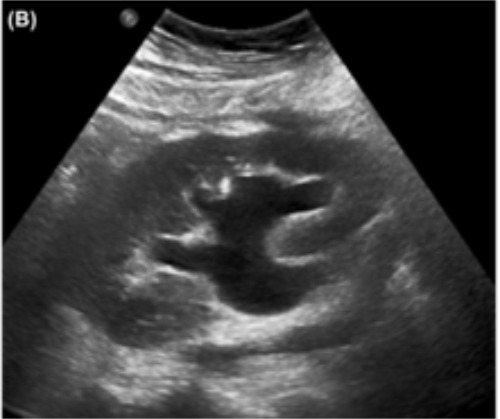
Moderate hydronephrosis. The usually hyperechoic sinus is pushed and thinned as the pelvis swells with hypoechoic urine, making the center of the kidney dark. Note the connection with the ureter, confirming that it is the urinary tract swelling and not parapelvic cysts.Image courtesy of Abhilash Koratala. renalfellow.org/pocus-gallery. Used with permission.

Ultrasonography can also assist in distinguishing AKI from chronic kidney disease (CKD). Often in CKD, the kidney length decreases, and severe CKD can be characterized by a combined kidney length of <20 cm 13. Changes in kidney size can also be indicators of other types of chronic disease. Enlargements in kidney size can be associated with deposition-type diseases, most notably amyloidosis, diabetes, lymphomatous invasion, and HIV 13, 14, 15, 16. In patients with amyloidosis and lymphoma with established kidney disease, changes in size help monitor the progression of disease or treatment response. Early in the progress of these diseases, there is deposition and swelling, but afterward, the kidney shrinks as in other progressive causes of CKD (Figure 1, Figure 2). 

Cortical thickness is another measurement to help distinguish CKD from AKI. Cortical thickness is measured on a longitudinal ultrasound image of the kidney, perpendicularly from the outer margin of the kidney to the corticomedullary junction. In a study by Beland et al. of 25 patients not on dialysis, the cortical thickness was shown to be more closely related to the estimated glomerular filtration rate (eGFR) than the kidney length. The normal thickness is between 8 and 10mm, Cortical thickness worsens in a stepwise progression with decreases in kidney function 17, 18. Cortical thickness also has excellent interobserver correlation, which makes it well suited for management and monitoring of kidney function over time 18, 19. 

Another characteristic of CKD is the degree of echogenicity. The echogenicity is the brightness of the cortex on ultrasound compared to the liver, assuming normal liver function. The kidney is normally isoechoic or hypoechoic to the normal liver or spleen (Figure 3B). One must have caution when making this comparison in patients with liver disease, as especially fatty liver disease or fibrosis can increase the echogenicity of the liver, making the comparison more difficult. 

**Figure 3:**
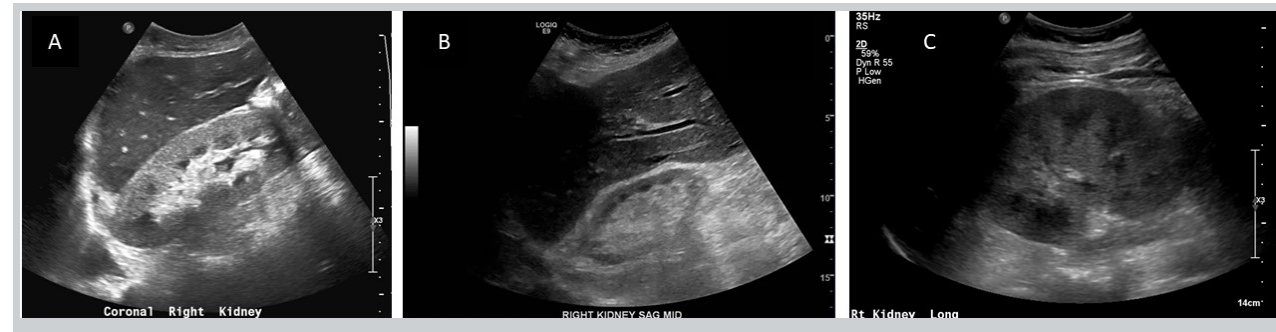
3. Increased Echogenicity. A) Right kidney long axis, hyperechoic cortex compared to liver, however cortex has normal thickness and length normal for size. Patient found to have positive serologies and proliferative lupus nephritis. B) Right kidney, long axis. Patient with advanced chronic kidney disease. Note the hyperechoic renal cortex and thin parenchyma with both cortex and medulla <1cm. C) Left kidney of patient with ATN, biopsy proven, from myoglobinuric kidney injury. Note the increased echogenicity of the cortex with increased differentiation of the medulla and cortex. Images courtesy of Abhilash Koratala Nephropocus.com Used with permission.

The echogenicity of the kidney is increased in disease states that increase fibrous tissue such as CKD. Inflammatory infiltrates from acute interstitial nephritis (AIN) or glomerulonephritis (GN) can also increase echogenicity due to inflammatory infiltrates, as will be discussed later. The amount of echogenicity correlates well with the severity of kidney disease and, when combined with decreased length, is specific for severe chronic kidney disease 13. A summary of etiologies of kidney dysfunction and their ultrasound findings are summarized in Table 2.

## AKI - Ultrasonographic Assessment

### Pre-renal AKI

AKI due to pre-renal etiologies requires the assessment of volume status in addition to metabolic and urinary data. The use of ultrasound in volume assessment is discussed in detail in this issue on POCUS, therefore will be reviewed briefly here. The volume state of patients is often difficult to assess in patients with extravasation of fluid due to cancer or liver disease or due to body habitus and so the true intravascular volume is unknown. Ultrasound of the inferior vena cava (IVC) and internal jugular vein can help to distinguish between fluid overload and volume depleted states 20, 21, 22. These measurements have improved interobserver reproducibility over jugular venous distension alone, and when combined have excellent correlation with the central venous pressure (CVP), especially when the volume status is low 21, 22. In this way, a point of care ultrasound can help guide the need for fluids in patients with AKI. 

### Post-renal AKI

Post-renal obstruction can occur acutely or can be a chronic clinical state. Special populations include children and men with prostate disease. CAKUT incidence in the United States is between 0.3-1.6 in 1000 births, compared to 4.2 per 10,000 births in Taiwan 23, 24, 25, 26. Benign prostatic hypertrophy is a more common entity in the US, with prevalence rates between 50%-75% among men older than 50 and 80% in men over 70 years old 27. Hydronephrosis is detected in <10% of patients evaluated for AKI, though this rate varies depending on the patient population (Figure 2) 28, 29. Hydronephrosis is described in non-pathologic physiologic states as well, most notably in pregnancy and congenital nephrogenic diabetes insipidus 30, 31, 32, 33, 34. Cases of non-dilated obstructive uropathy have been reported in patients with malignancy, nephrolithiasis, and post-surgical collections which lead to kidney parenchymal compression and high pressure in Bowman's space mitigating the development of hydronephrosis 35, 36, 37, 38. Of particular importance is to rule out obstruction in patients with malignancy, as obstruction can sometimes be the presenting complaint in progression of disease. Indeed, any part of the urinary tract can be affected by malignancy and should be monitored closely when a history of malignancy is present 39.

**Figure 4:**
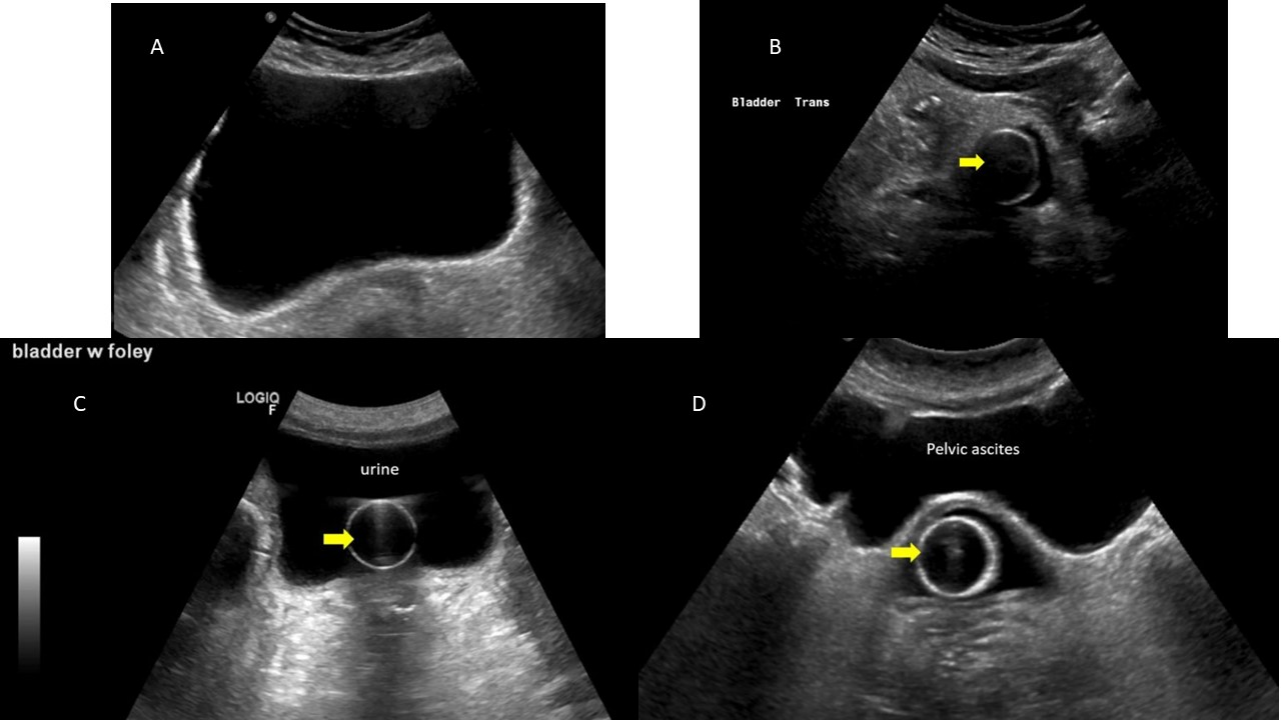
Bladder ultrasound. A) Normal transverse ultrasound of a full bladder. B) Decompressed bladder around an indwelling urinary catheter. Notice a small amount of hypoechoic urine around the catheter which is normal. C) Indwelling urinary catheter within a non-empty bladder. Note there is still a lot of anechoic urine within the bladder. This measured as >200 mL indicating improper drainage of urine. The indwelling urinary catheter was replaced and urinary flow resumed. D) Pelvic ascites mimicking bladder. Ascites is a common false positive for bladder scanners which mistake it for urine. On ultrasound we see the indwelling urinary catheter surrounded by a decompressed bladder (arrow). Superficial to this, one can see pelvic ascites interdigitating among abdominal viscera and tracking into the paracolic gutters. Images courtesy of Abhilash Koratala. Renalfellow.org/pocus-gallery. Used with permission.

Another consideration that contributes to diagnoses of hydronephrosis includes appropriate interpretation of echogenicity of kidney and evaluation of the bladder wall (Figure 4). Visualizing the distal ureter at the ureteropelvic junction and the proximal ureter at the junction with the renal pelvics can provide clues in evaluating the etiology of hydronephrosis. Central sinus fat is normally echogenic. Kidney calyces are not visible unless dilated and hydronephrosis should be differentiated from a kidney sinus cyst which is rounded and has no communication with the collecting system. Stones, masses, and prostatic hypertrophy can often be identified as etiologies of obstruction in those with hydronephrosis. Within the bladder, color Doppler ultrasound can identify ureteral jets which can help distinguish functional (presence of jets) and non-functional (absence of jets) obstruction 40. Ultrasound of the indwelling urinary catheter in the bladder ensures appropriate placement.

Papillary necrosis is an interesting cause of hydronephrosis. The necrosed papilla sloughs into the urinary space and fills the ureter at the site of obstruction 41. Papillary necrosis results due to ischemic changes at the kidney pyramids, which are more vulnerable to ischemia due to low blood flow and a hypertonic environment. This can be related to sickle cell disease, sickle cell trait, analgesic abuse, diabetes, vesicoureteral reflux, or other vasculitic diseases. In cases where the necrosed papillae remain in situ, or with repetitive events such as with sickle cell disease, the papillae can become calcified, which can be readily seen on kidney ultrasound. 

### Intrinsic AKI

The broadest and most varied cause of AKI has to do with the kidney itself, and ultrasound can offer glimpses into diagnosis and treatment in this case as well. AIN is most readily diagnosed by biopsy, but ultrasound can play a role as well. HIV associated nephropathy has been studied and followed before effective treatment and was found to have increased echogenicity in the acute phase, and the amount of echogenicity and response to treatment correlated well with survival 42, 43. This has also been applied to lupus nephritis and other causes of AIN to monitor the response to treatment and predict flares. As with other types of kidney disease, later in the time course the echogenicity becomes permanently increased and the size of the kidney is reduced. 

Diabetic nephropathy can have characteristic findings on ultrasound. During the early phase of diabetic nephropathy, the kidney is enlarged with a concurrent rise in GFR associated with hyperfiltration 16. This is seen concurrently with microalbuminuria, and as the disease progresses it will then start to sclerose 44. In patients with diabetes mellitus and advancing CKD based on eGFR with “normal” kidney size, the kidney size may be misleading as diabetic kidney disease can lead to enlargement of the kidneys. In these patients, this phase of diabetic kidney disease is often rapid and will fit the definition of AKI, though this actually reflects ongoing CKD progression. 

One of the most common causes of AKI in the ICU is acute tubular necrosis (ATN). ATN is caused by a variety of conditions, including the progression of pre-renal and post-renal injuries, sepsis, shock, and both endogenous and exogenous toxins. On ultrasound, there is decreased mobility with respiration, enlargement, and a thickened capsule with impaired corticomedullary differentiation 45. Early in ATN, the increased echogenicity tends to spare the medullary pyramids, yielding greater corticomedullary differentiation (Figure 3C). However, as it progresses or becomes more severe, the medullary pyramids become swollen and echogenic as well due to distal tubule involvement 30, 45. Monitoring the size of the kidney during ATN can help to prognosticate as well, with increased anteroposterior to longitudinal diameter ratio having a worse prognosis 46, 47.

Infiltrative disease can be readily monitored and assessed on ultrasound as already mentioned. While serologies and biopsy are the mainstays of diagnosis, monitoring and differentiation can be readily assessed with repeated kidney ultrasound. In multiple myeloma, the kidney will appear hyperechoic with no change in kidney size and no change in resistive index (RI) 48. In lymphoma, the kidney often appears hypoechoic and diffusely enlarged on ultrasound 49. One should take particular care to differentiate this imaging from xanthogranulomatous pyelonephritis, which should be readily distinguished by the presence of staghorn calculus and pyuria in the latter. Amyloid, like multiple myeloma, will typically demonstrate increased cortical echogenicity on ultrasound, but is differentiated clinically by the presence of albuminuria and nephrotic synrome, rather than paraproteinuria. AA amyloidosis can be associated with chronic inflammation or chronic infections, such as tuberculosis 50. The amyloid kidney is often enlarged early in the disease course and, like diabetes, becomes smaller with loss of kidney function 15. 

### Vascular assessment

Point of care ultrasound with Doppler assessment of the abdominal parenchymal vessels has found increasing use in recent years. It is a higher level application of B-mode point of care ultrasound and is discussed separately to differentiate from the general discussion of point of care ultrasound in AKI, but many of the assessments can be used to confirm diagnosis as above. 

The resistance index (RI) is defined as the (peak systolic velocity – end-diastolic velocity)/peak systolic velocity of the intrarenal arteries. Normal RI is considered between 0.5-0.7, with elevations associated with higher ICU mortality and persistent AKI 51. Most parenchymal diseases result in a non-specific elevation, such as obstruction, hepatorenal syndrome, sepsis, and in acute rejection in the transplant allograft. It is normal to decreased in pre-renal azotemia, however, and may be useful in distinguishing pre-renal vs parenchymal disease/ATN when <0.7 52. 

The venous excess ultrasound (VExUS) grading system was developed to help increase the ability to distinguish vascular overload and organ congestion. It is discussed in detail elsewhere, so will be mentioned briefly here. In a post hoc analysis of a single-center prospective study of 145 patients, Beaubien-Souligny et al. created a system to grade the ultrasound findings combining IVC measurement, hepatic vein Doppler, portal vein Doppler, and intra-renal venous Doppler. This combination, used in “VExUS C” grading system outperformed all others including CVP measurement and IVC measurement alone to predict AKI 53. This finding was in non-critically ill patients undergoing cardiac surgery, and so may not be able to be generalized to other patients. When there is a systolic reversal of hepatic venous flow, severe portal flow pulsatility, severe alteration in intra-renal venous flow, and expanded IVC there is a high likelihood of venous congestion 53, 54.

Nephrotic syndrome is a disease state where vascular ultrasonography for volume status assessment is of particular importance. Patients with nephrotic syndrome are hypercoagulable from several different mechanisms but have been found to have an increased risk of venous thromboembolism, and about 40% of patients develop some form of clot 55. When these patients develop AKI, it is important to rule out renal vein thrombosis as the cause, as there is a high rate among patients with nephrotic syndrome 56. 

The last aspect of the vascular exam in AKI is a consequence of impaired flow, which Doppler ultrasound can confirm. Renal infarction is often embolic and sequelae of cardiac disease as the main source of embolic material. The infarct is an echo-free area, with swollen parenchyma, and Doppler can be used to confirm the lack of flow to the area 57. This should be contrasted to cortical necrosis, which is a dark hypoechogenic kidney cortex entirely and is secondary to severe ischemia due to vascular abnormalities such as pregnancy with eclampsia, severe sepsis, pancreatitis, or other severe shock state 58. 

**Figure 5:**
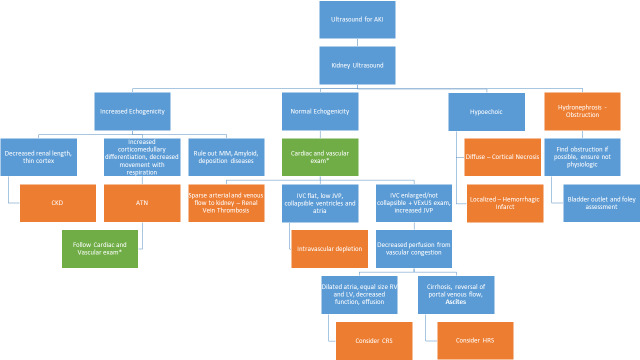
Flowsheet on the approach to AKI and ultrasound. Using this flowsheet and starting with a kidney ultrasound, one can decipher most causes of AKI.

The combination of renal and body ultrasound can help to distinguish different types of AKI, as described in Figure 5. This stepwise approach can help providers methodically investigate AKI from the context of ultrasound and hopefully provide improved patient care. 

## Future directions of ultrasound

### Ultrasound as a therapeutic

Ultrasound has long been used by physical therapy as a modality to improve local swelling and inflammation. The application of ultrasound alone appears to have postinjury modulation and prevents inflammation in the kidney by stimulating the splenic cholinergic anti-inflammatory pathway 59. Indeed, it has been shown in mice to markedly attenuate kidney ischemia-reperfusion injury 60. While these studies were performed in mice and have not been validated in humans, the use in muscle and joints is well established in the physical therapy community and is likely to be applicable in humans.

### Contrast-enhanced Ultrasound

Contrast for ultrasound has been used to enhance image intensity from the vasculature. It can give more information on kidney perfusion and microvascular function as well as help localize injury. The newest forms of contrast are gas-filled microbubbles that are surrounded by a shell that is quickly metabolized and the gas exhaled. It is an attractive option as it is non-toxic, does not expose the patient to radiation, and can be done at the bedside. It does require proprietary contrast-specific software which can inhibit its use in resource-limited areas. It has been used to better image lesions in the kidney and to predict acute rejection in transplant patients 61, 62. Another intriguing possible use is targeted contrast agents with disease-specific antibodies using the signal intensity as correlation with the extent of injury or disease 63. These disease-specific microbubbles are also being looked at to deliver medications to local sites via locally increased permeabilization 59. Further translational research is needed, but the outlook is currently promising. 

**Table 2:** Summary of ultrasound findings in kidney insufficiency.

**Kidney Insufficiency**	**Ultrasound Findings**	**Pearls and Pitfalls**	**Next steps**
**CKD**	Decreased kidney length 8, decreased cortical thickness 17, 18, Increased echogenicity 13	Increased echogenicity is subjective and can be skewed by liver disease. Depending on disease course kidney length can be increased or decreased.	Establish baseline serum creatinine, monitor for change, rule out AIN
**Pre-Renal AKI**	Decreased vascular exam (low IVC collapsibility index, low ultrasound internal jugular collapse point, collapsible ventricles, and atria), clear lung fields	Mixed pictures are often possible in disease states such as pulmonary hypertension and liver cirrhosis with high total body or lung fluid and low intravascular fluid	Fluid resuscitation, amplification of forward flow with pressors or inotropes, monitoring of creatinine change
**Post-Renal AKI**	Hydronephrosis, calcified papillae	Non-dilating obstruction also exists, if high suspicion may need functional test or urologic procedure 35, 36, 37, 38 Non-pathologic causes of hydro: pregnancy, with high amounts of diuresis, and with congenital nephrogenic diabetes insipidus 30, 31, 32, 33, 34	Relief of obstruction, monitoring of creatinine change, treatment of underlying papillary necrosis
**AIN**	Increased echogenicity	Multiple causes with varied workup. Echogenicity non-diagnostic	Kidney biopsy, serologic markers, monitor echogenicity and size for improvement with treatment
**DM**	Increased size, then decreased size with sclerosis	Normal kidney size can be an indication of entering sclerosing phase	Aggressive treatment and monitoring of DM
**ATN**	Decreased mobility with respiration, enlargement, thickened capsule, ±corticomedullary differentiation 45	Early ATN: increased corticomedullary differentiation.Late/severe ATN: decrease corticomedullary differentiation 30, 45	Treat the underlying cause, monitor anteroposterior to longitudinal diameter ratio: increased having a worse prognosis 46, 47
**Infiltrative disease**	Multiple myeloma: hyperechoic, no change in size 48 Lymphoma: hypoechoic, and diffusely enlarged 49 Amyloid: hyperechoic, enlarged early then small	Differentiate lymphomatous invasion from xanthogranulomatous pyelonephritis	Kidney biopsy, serologies, monitor size and echogenicity for treatment response
**Venous Congestion**	Systolic reversal of hepatic venous flow, severe portal flow pulsatility, severe alteration in intra-renal venous flow, and expanded IVC with low collapsibility index. High ultrasound collapse point on internal jugular vein	Many alterations in intraabdominal pressure (ascites, surgical abdomen), intrathoracic pressure (Intubation, pulmonary hypertension) can affect the individual measurements and should be taken as a whole	Diuresis, serial monitoring of vascular findings to confirm improvement with fluid removal
**Renal vein thrombosis**	Sparse arterial and venous flows, reverse diastolic flows, increased resistive index 64	Important in the context of nephrotic syndrome	Anticoagulation, consider invasive treatment
**Hemorrhagic infarct**	Hypoechoic free area, swollen parenchyma, lack of arterial flow to the area	Almost always secondary to cardiac disease as a source of embolism	Treatment of underlying disease
**Cortical Necrosis**	Hypoechogenic cortex	Only seen in severe ischemic states	Treatment of underlying shock, poor prognosis

## Case example 1

**Figure 6:**
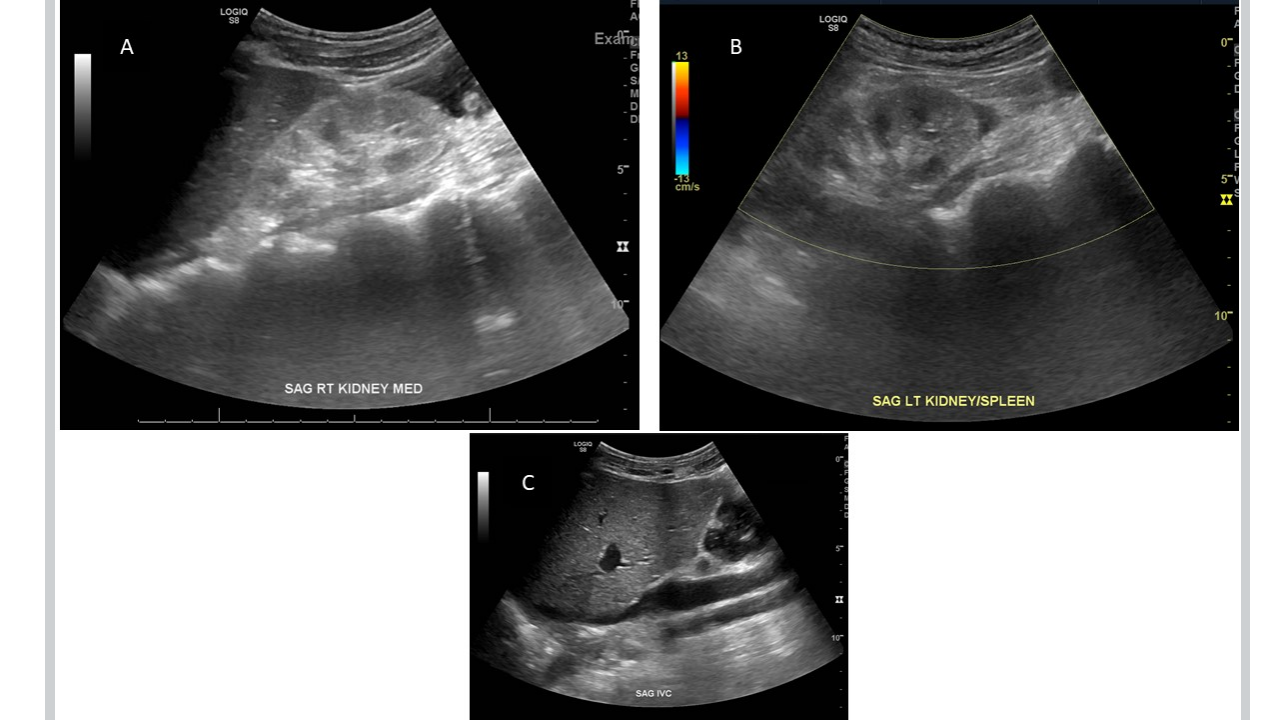
Bilateral Kidney and IVC: Case 1. A) The right kidney appears more echogenic compared with the adjacent liver and has increased medullary pyramid differentiation consistent with ATN. B) Left kidney also with increased echogenicity and differentiation. C) Liver, IVC, and aorta in view. IVC collapsibility index >50%. JVP on ultrasound also found to be at the level of the clavicle (not shown).

Patient is a 30-year-old male with a past medical history of familial intrahepatic cholestasis and a liver transplant 21 years prior. He has CKD presumed secondary to calcineurin inhibitor use with a baseline creatinine of 1.3-1.5 mg/dL. He also has chronic pancreatitis, developmental delay, and malabsorption with a PEG tube. He presents to your care post-procedure after a failed clipping of a gastric fistula. Following the gastric fistula procedure, the patient was found to have AKI with creatinine rise from baseline to 6.06 mg/dL. The patient’s physical exam is normal except for prior PEG tube site with clean and dry bandage overlying. Vital signs are within normal limits. On review of medication and discussion with patient, no nephrotoxins are identified. Kidney ultrasound is performed (Figure 6) and the radiologist’s reading notes bilateral echogenic and atrophic kidneys, likely related to chronic kidney parenchymal disease without hydronephrosis or nephrolithiasis, presumed to be progression of disease. On review by the nephrologist, the kidney cortex is not diminished and there is increased differentiation of the medulla to the cortex, with kidney length likely within in the normal limits of this smaller adult. Given this, a vascular exam was performed which showed multiple signs of intravascular volume depletion, including an IVC with collapsibility index >50%, JVP at the clavicle, and clear lung exam without B-lines. This was consistent with a history of gastrointestinal losses with likely occult hypotension resulting in AKI from pre-renal volume depletion and hypotension. The patient was volume resuscitated, but continued to have a rise in creatinine to a peak of 12.49 mg/dL. Lack of response to volume challenge indicated that the patient had been volume deplete for some time, ATN had likely occurred from prolonged kidney ischemia. However, his kidney function recovered back to baseline once ongoing gastrointestinal losses stopped and intravascular fluid was successfully repleted. 

Ultrasound is currently underutilized in AKI. It is not only able to diagnose obstruction, but it can also yield important data on underlying CKD status, vascular status, ATN, and inflammatory states of the kidney. The most important aspect of ultrasound is its ability to be performed quickly at the bedside throughout the hospital stay, helping to monitor response to therapeutics and aid in diagnosis as changes occur. The ultrasound itself may eventually be used as a therapeutic and contrast-enhanced ultrasound can both enhance diagnosis and may be used to deliver therapeutics as well. It has become an invaluable tool in the clinician’s tool kit. 

## Disclosures

None
